# *Anteon hubeni* a new species from Ecuador (Hymenoptera: Dryinidae)

**DOI:** 10.3897/BDJ.8.e56613

**Published:** 2020-09-28

**Authors:** Massimo Olmi, Mario Contarini, Leonardo Capradossi, Adalgisa Guglielmino

**Affiliations:** 1 Tropical Entomology Research Center, Viterbo, Italy Tropical Entomology Research Center Viterbo Italy; 2 University of Tuscia, Viterbo, Italy University of Tuscia Viterbo Italy

**Keywords:** Anteoninae, *Anteon oliveirai*, Chrysidoidea, Neotropical, key

## Abstract

**Background:**

*Anteon* is a speciose genus widespread in all zoogeographical regions, except Antarctica.

**New information:**

A new species, *Anteon hubeni* sp. n., is described below from Ecuador, Pichincha Province. The new species is similar to *Anteon oliveirai* Olmi, known from Brazil, Minas Gerais. The main difference between these two species concerns the sculpture of the frons: in *A. oliveirai*, the frons shows two lateral keels around the orbits directed towards the antennal toruli, whereas in *A. hubeni*, these keels are not present. The key to the Neotropical species of *Anteon* is modified to include the new species.

## Introduction

Dryinidae (Hymenoptera: Chrysidoidea) from Ecuador have been studied mainly by Olmi and Virla (2014) in a monograph on the Neotropical Dryinidae. With 464 world species (Olmi et al. 2019) and 72 in the Neotropical region (Olmi and Virla 2014), the genus *Anteon* Jurine, 1807, is the largest in the subfamily Anteoninae. Species of *Anteon* are known to parasitise various leafhoppers belonging to Cicadellidae, many of which are significant pests of cultivated plants (Guglielmino et al. 2013). In 2019, we received on loan a small collection of unidentified Dryinidae collected in Ecuador by Mike Huben, an independent researcher working for many years in that country. Amongst this material, there was a new species of *Anteon* described below.

## Materials and methods

The description follows the morphological terminology of Olmi and Virla (2014), partly updated following Azevedo et al. (2018), Kawada et al. (2015), Lanes et al. (2020). The measurements reported are relative, except for the total length (head to metasomal tip, without antennae and sting). Antennal proportions refer to the lengths of the relevant segments as proportions of each other, values rounded to the nearest whole number. The following abbreviations are used: POL, distance between the inner edges of the two lateral ocelli; OL, shortest distance between the edge of a lateral ocellus and the median ocellus; OOL, distance from the outer edge of a lateral ocellus to the compound eye; OPL, distance from the posterior edge of a lateral ocellus to the occipital carina; TL, distance from the posterior edge of the eye to the occipital carina.

The types of all Neotropical species of *Anteon* have been previously examined by the authors.

The specimens studied in this paper are deposited in the following collections: QCAZ: Museo de Zoologia, Sección Invertebrados, Pontificia Universidad Católica del Ecuador (PUCE), Quito, Ecuador; DBUSU: Department of Biology, Utah State University, Logan, Utah, USA.

The description of the new species is based on a single specimen. The authors are aware that descriptions of new taxa should normally be based on more than one individual. However, the Dryinidae are so scarce that more than one specimen of each species can be rarely procured. In addition, on the basis of our experience and knowledge, species are sufficiently delimited by unique characters to justify their description.

## Taxon treatments

### Anteon hubeni

Olmi, Contarini, Capradossi, Guglielmino, 2020 sp. n.

0931F3F6-B792-5C02-B04B-811CC335834E

74F38422-FA72-406C-A233-16D7B023D65A

#### Materials

- **Type status:** Holotype. **Occurrence:** catalogNumber: QCAZ3280; recordedBy: M. Huben; individualCount: 1; sex: female; **Taxon:** order: Hymenoptera; family: Dryinidae; genus: Anteon; specificEpithet: hubeni; taxonRank: species; **Location:** continent: South America; country: Ecuador; locality: between Pifo and Papallacta; verbatimElevation: 3431 m; verbatimLatitude: 00°15.56'S; verbatimLongitude: 78°14.48'W; **Identification:** identifiedBy: Massimo Olmi; Mario Contarini; Leonardo Capradossi; Adalgisa Guglielmino; **Event:** samplingProtocol: Yellow Pan Trap; eventDate: 27 August–9 September 2018; **Record Level:** type: physical object; institutionID: Museo de Zoologia, Sección Invertebrados, Pontificia Universidad Católica del Ecuador, Quito, Ecuador; collectionCode: QCAZ; basisOfRecord: Preserved specimen

#### Description

**Figure 2.**
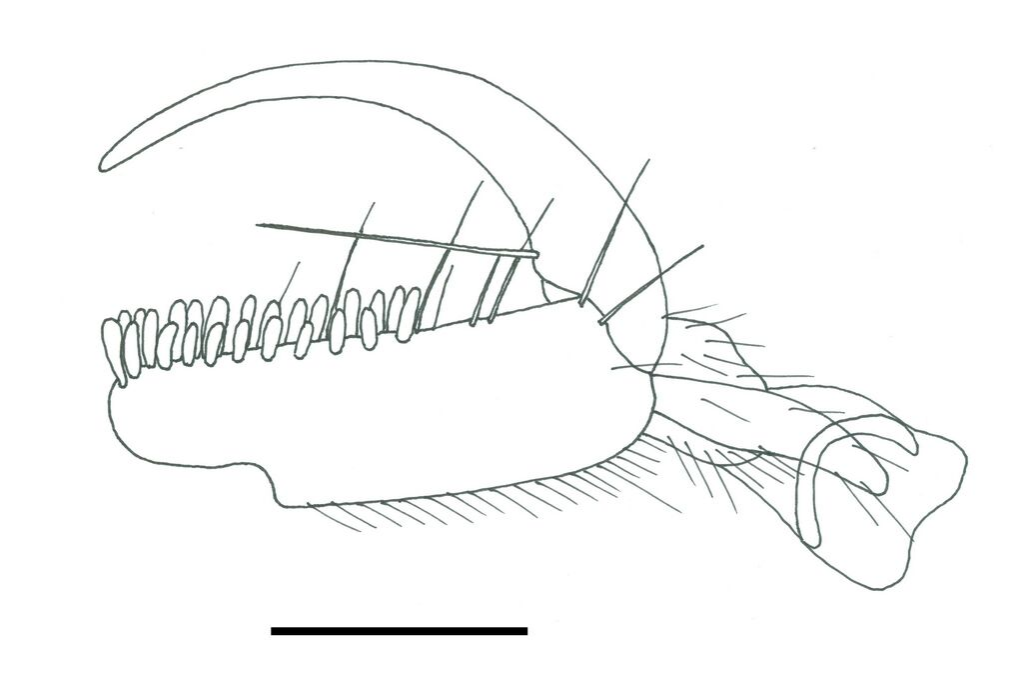
Chela of holotype of *Anteon hubeni*, sp. n. Scale bar = 0.1 mm

Female. Fully winged (Fig. 1a, b); length 3.3 mm. Head black, except mandible testaceous; antenna brown, except antennomeres 4–6 and part of 7 testaceous; mesosoma black; metasoma brown; legs testaceous. Antenna clavate; antennal segments in following proportions: 11:7:9:7:6:6:6:6:6:7. Head (Fig. 1c) shiny, with frons unsculptured; vertex behind ocelli slightly granulate, between ocelli slightly rugose, without two tracks of oblique keels connecting posterior ocelli to occipital carina; frons without two lateral keels around orbits directed towards antennal toruli (Fig. 1c); frontal line incomplete, only present in posterior half of frons; occipital carina complete; POL = 7; OL = 3; OOL = 7; OPL = 5; TL = 7; greatest breadth of posterior ocelli about as long as OL. Pronotum shiny, with strong transverse impression between anterior and posterior surface; anterior part of posterior surface without raised transverse carina; posterior surface shiny, sculptured by transverse keels and posteriorly punctate, shorter than scutum (5:20); pronotal tubercle reaching tegula. Mesoscutum (Fig. 1a) shiny, unsculptured, partly very slightly granulate. Notauli incomplete, reaching approximately 0.5 × length of mesoscutum. Mesoscutellum and metanotum shiny, smooth, slightly punctate, unsculptured between punctures. Metapectal-propodeal complex reticulate rugose, with strong transverse keel between disc and propodeal declivity; propodeal declivity without longitudinal keels (Fig. 1a). Forewing hyaline, without dark transverse bands; distal part of stigmal vein (2r-rs&Rs) shorter than proximal part (4:9). Protarsomeres in following proportions: 8:2:3:4:12. Protarsomere 5 (Fig. 2) with basal part much longer than distal part. Enlarged claw (Fig. 2) with proximal prominence bearing one long bristle. Protarsomere 5 (Fig. 2) with inner side straight, with two rows composed of approximately 25 lamellae, without interruption to distal apex. Tibial spurs 1/1/2.

#### Diagnosis

Female of *Anteon* with frons not provided with lateral keels around orbits directed towards antennal toruli (Fig. 1c), mesoscutum mostly unsculptured (Fig. 1a), propodeal declivity not provided with longitudinal keels (Fig. 1a), protarsomere 1 twice as long as protarsomere 4.

**Figure 3.**
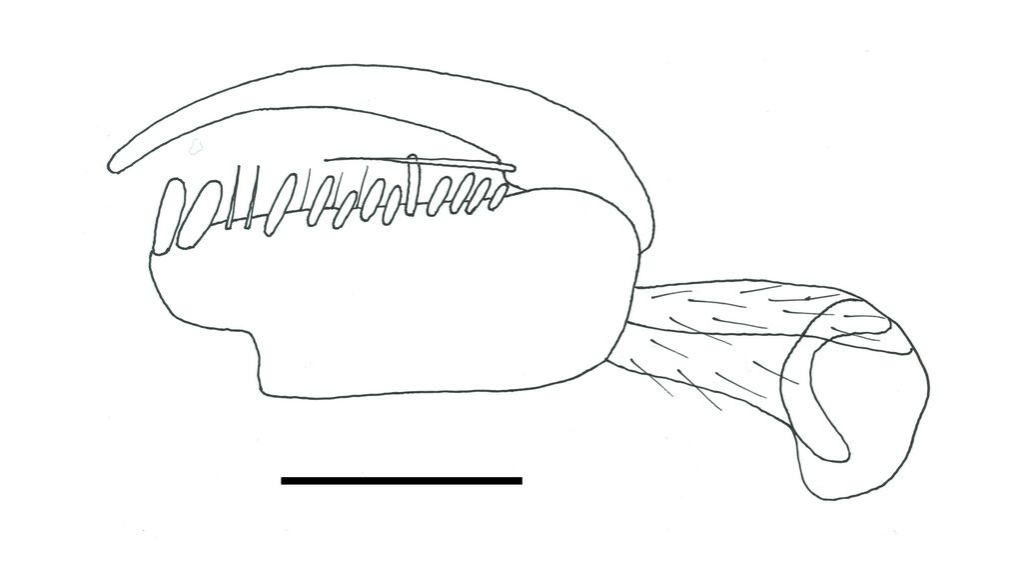
Chela of holotype of *Anteon oliveirai* Olmi. Scale bar = 0.2 mm

Based on the characters indicated above, *A. hubeni* sp. n. is close to *A. oliveirai* Olmi 1984, known from Brazil (female holotype No. 1625 from Minas Gerais, Serra do Caraça, I.1970, F.M. Oliveira leg. (originally in the American Entomological Institute, Gainesville, Florida, now relocated in DBUSU)) (Figs 3, 4). However, in *A. oliveirai*, the frons shows two lateral keels around the orbits directed towards the antennal toruli (Fig. 4d), whereas in *A. hubeni*, these keels are not present (Fig. 1c). Following the above description of the new species, the key to the females of the Neotropical *Anteon* species published by Olmi and Virla (2014) (not reproduced entirely here, because it is composed of 44 couplets) can be modified by replacing the first five couplets as below.

#### Etymology

The species is named after the collector, Dr. Mike Huben.

#### Distribution

The new species is known only from the type locality.

#### Ecology

The new species was collected in a *Polylepis* forest.

## Identification Keys

### Part of the key to the females of the Neotropical *Anteon* species published by Olmi and Virla (2014) including the new species 

**Table:** 

1	Protarsomere 4 at most 0.5x as long as protarsomere 1	2
–	Protarsomere 4 as long as, or only slightly shorter or longer than, protarsomere 1	Couplets from 13 to 44
2	Propodeal declivity with two longitudinal keels	3
–	Propodeal declivity without longitudinal keels (Figs 1a, 4a)	5
3	Pronotum forming two dorsal lobes near posterior margin	*A. topali* Olmi
–	Pronotum not forming two dorsal lobes near posterior margin	4
4	Protarsomere 5 with basal part about as long as distal part (Plate 39D in Olmi and Virla 2014); frons sculptured by numerous longitudinal keels	*A. perniciosum* Olmi
–	Protarsomere 5 with basal part much longer than distal part (Plate 23B in Olmi and Virla 2014); frons smooth, not sculptured by numerous longitudinal keels	*A. catarinense* Olmi
5	Mesoscutum slightly punctate, unsculptured between punctures (Figs 1a, 4c)	5'
–	Mesoscutum completely strongly or slightly granulated, occasionally partly rugose or sculptured by irregular keels	Couplets from 6 to 12
5'	Frons with two lateral keels around orbits directed towards antennal toruli (Fig. 4d)	*A. oliveirai* Olmi
–	Frons without lateral keels around orbits directed towards antennal toruli (Fig. 1c)	*A. hubeni* sp. n.

## Discussion

Olmi and Virla (2014) recorded 72 species of *Anteon* from the Neotropical region, amongst which are 19 from Ecuador (area: 283,561 km^2^). Following the above description, this number increases to 20, more than in the closest countries, Colombia (area: 1,141,748 km^2^) and Peru (area: 1,285,220 km^2^), where eleven and seven species are known, respectively (Olmi and Virla 2014). The difference is probably connected with more research undertaken in Ecuador.

## Supplementary Material

XML Treatment for Anteon hubeni

## Figures and Tables

**Figure 1a.**
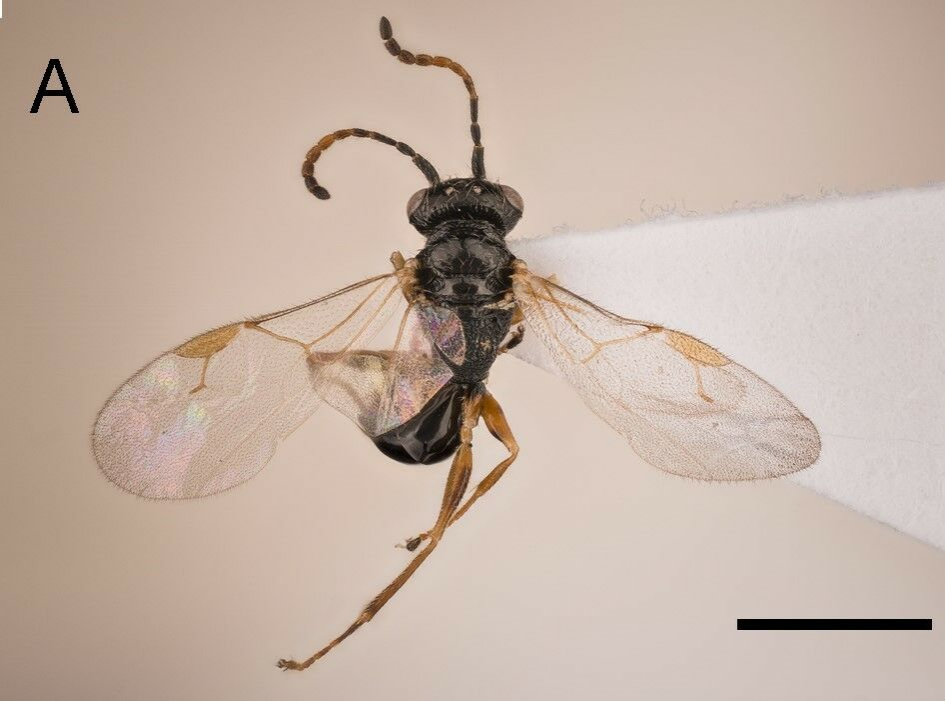
Habitus in dorsal view. Scale bar = 3.0 mm

**Figure 1b.**
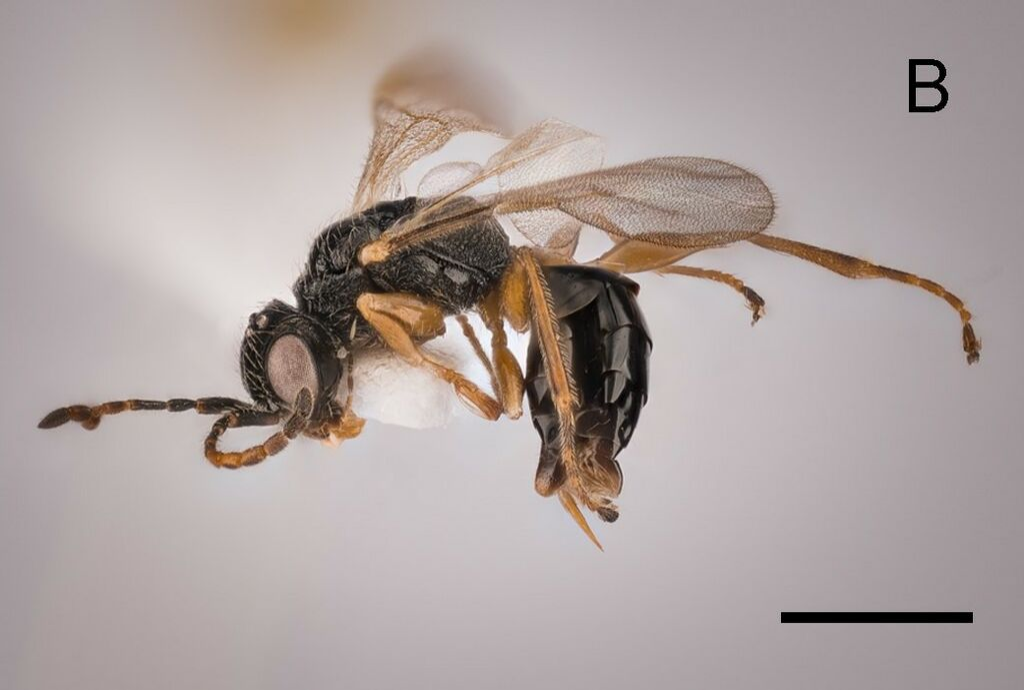
Habitus in lateral view. Scale bar = 1.6 mm

**Figure 1c.**
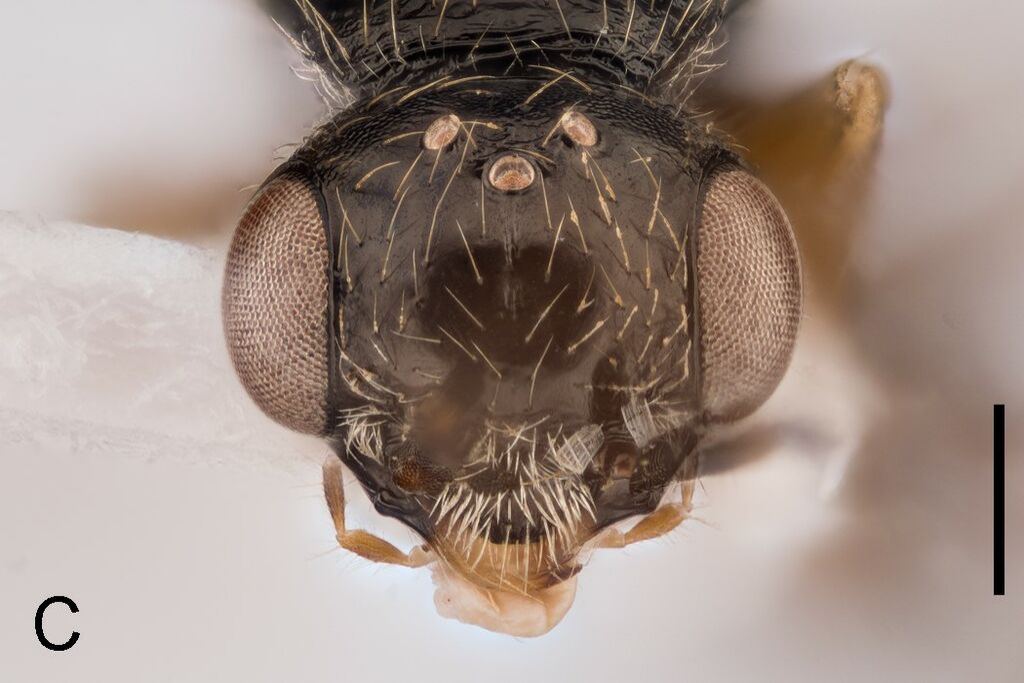
Head in frontal view. Scale bar = 0.3 mm

**Figure 4a.**
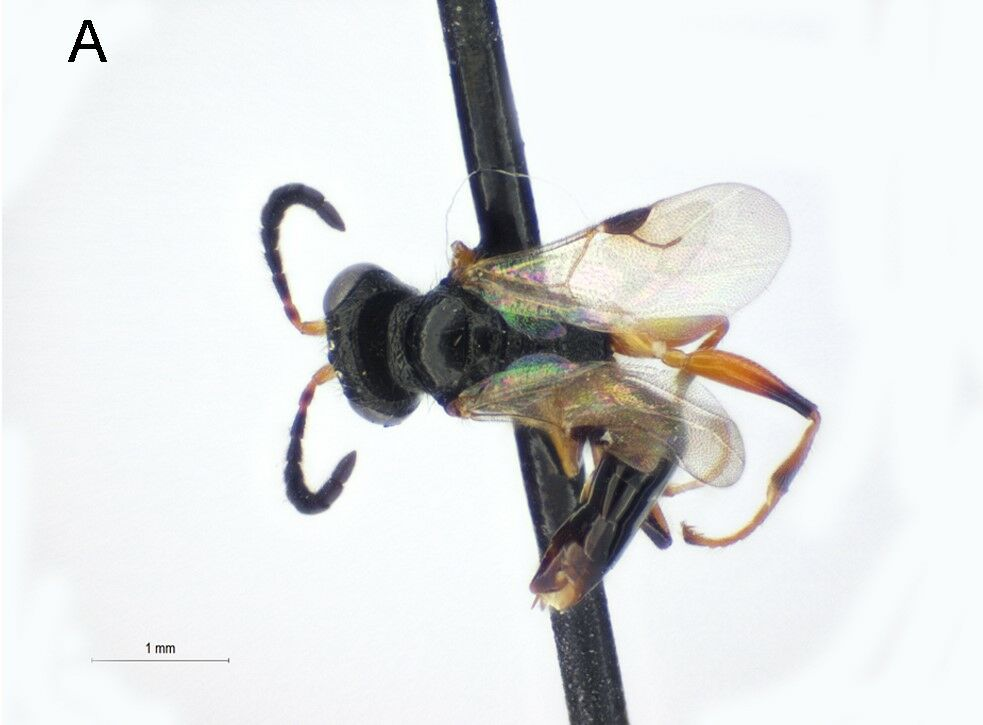
Habitus in dorsal view

**Figure 4b.**
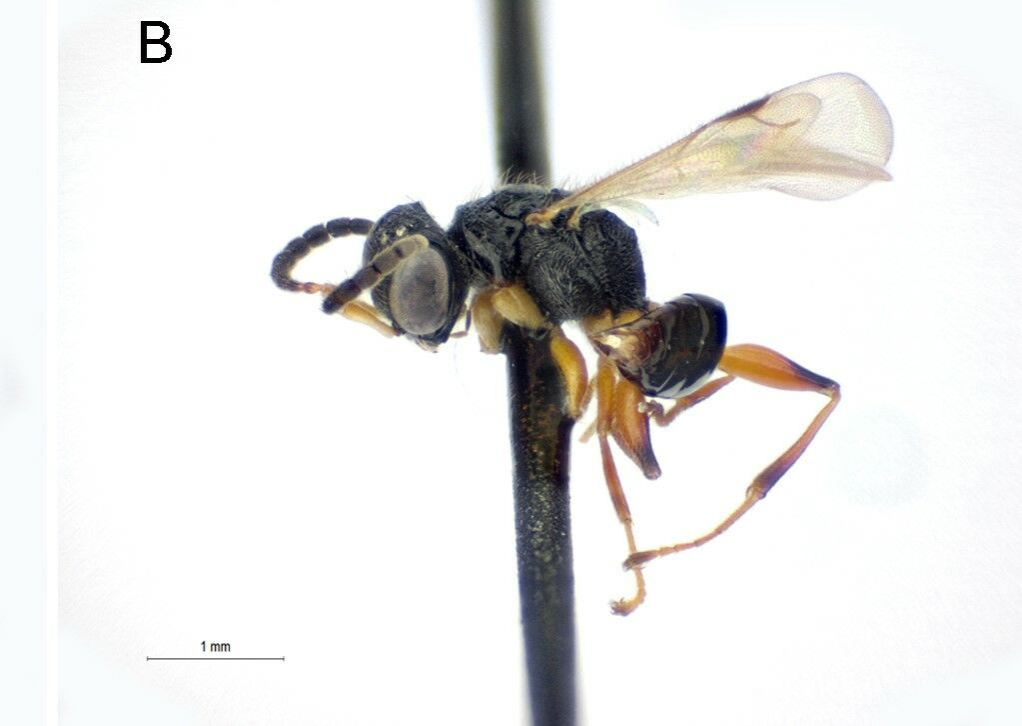
Habitus in lateral view

**Figure 4c.**
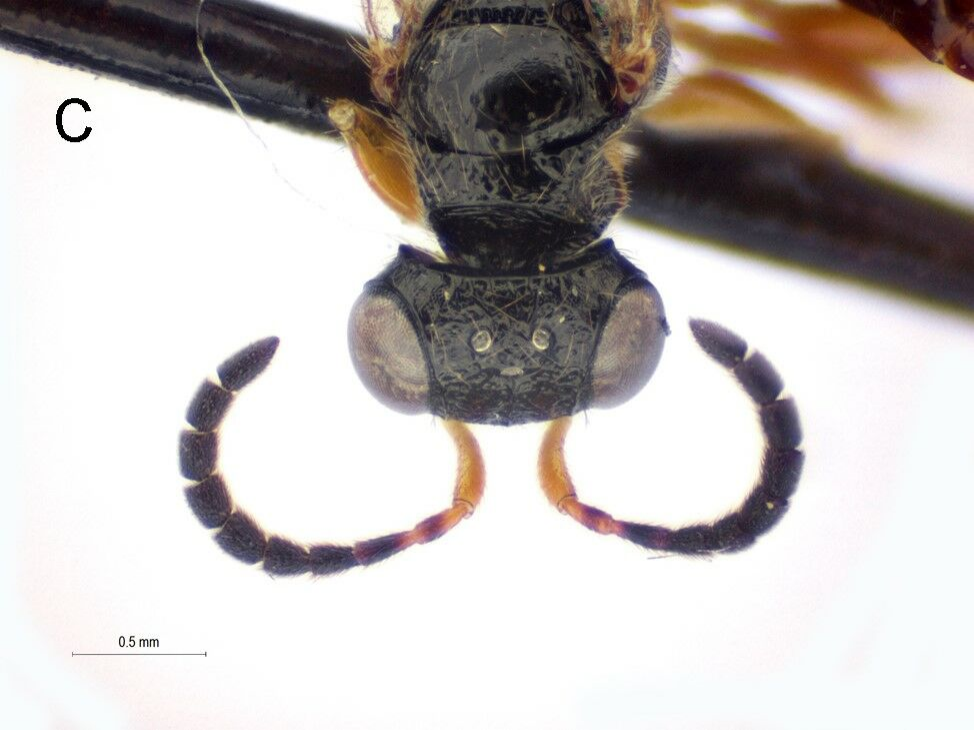
Head, pronotum and mesoscutum in dorsal view

**Figure 4d.**
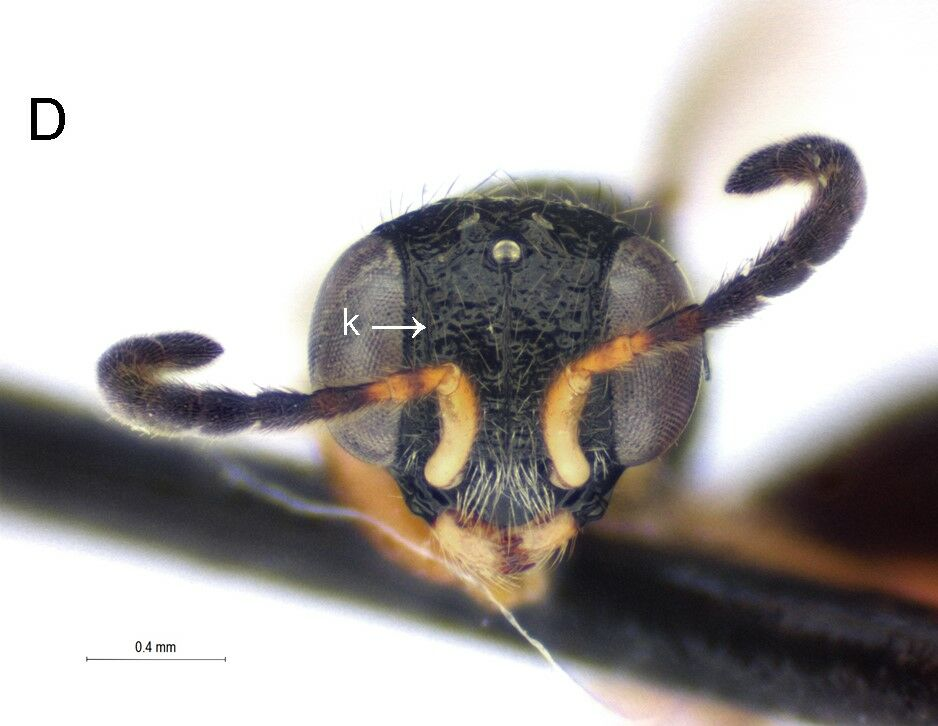
Head in frontal view. k = lateral keel along orbit.
